# Concept Mapping, an Effective Tool for Long-Term Memorization of Anatomy—A Quasi-Experimental Research Carried out among 1st Year General Medicine Students

**DOI:** 10.3390/ejihpe10010038

**Published:** 2020-03-03

**Authors:** Sergiu-Mihai Nicoara, Stefan-Emeric Szamoskozi, Delia-Alexandrina Mitrea, Daniel-Corneliu Leucuta

**Affiliations:** 1 Department of Human Anatomy and Embryology, Faculty of General Medicine, Iuliu Hatieganu University of Medicine, 400012 Cluj-Napoca, Romania; 2 Department of Applied Psychology, Faculty of Psychology and Educational Sciences, Babes-Bolyai University, 400029 Cluj-Napoca, Romania; istvan.szamoskozi@ubbcluj.ro; 3 Computer Science Department, Technical University, 3400 Cluj-Napoca, Romania; delia.mitrea@cs.utcluj.ro; 4 Department of Medical Informatics and Biostatistics, Faculty of General Medicine, Iuliu Hatieganu University of Medicine, 400012 Cluj-Napoca, Romania; dleucuta@umfcluj.ro

**Keywords:** concept formation, concept map, learning, long-term memory, language, age, learning curve

## Abstract

This study is part of a doctoral thesis conducted at the Faculty of Psychology of Babes-Bolyai University in collaboration with the University of Medicine, both from Cluj-Napoca, Romania. The starting point of the study was based on the eternal question of the medical student—“How should I learn to manage to retain so much information?” This is how learning through conceptual maps and learning by understanding has been achieved. In the study, a number of 505 students from the Faculty of General Medicine were randomly selected and divided into groups, to observe changes in the grades they obtained when learning anatomy with the concept mapping method vs. traditional methods. Six months later, a retest was carried out to test long-term memory. The results were always in favor of the experimental group and were statistically significant (with one exception), most notably for the 6-month retesting. It was also observed that the language of teaching, different or the same as the first language, explains that exception, at least partially. Other results were taken into account, such as the distribution of bad and good grades in the two groups. Other parameters that influenced the obtained results and which explain some contradictory results in the literature are discussed. In conclusion, the use of conceptual maps is useful for most students, both for short and long-term memory.

## 1. Introduction

We started from the idea that learning by using conceptual maps improves the memorization of anatomical notions in the short term but it was desirable to see if this happens in the long term as well. The present research had as a starting point the need of the student to accumulate new information and skills, which can, at one point, become a hindrance to cognitive development due to their volume and complexity. Basically, we merely placed ourselves in the skin of the students we once were, to see what we would like our current teacher to teach us. Thus, we noticed that beyond the need for accumulation, which up to a certain point can be dealt with, there is also the need to put together all this knowledge and skills, to observe the connections between them and how they can be used in a new situation. We somehow merged the present and the past in an attempt to generate an idea for the future.

The present paper is an integral part of a doctoral study of Babes-Bolyai University, Faculty of Psychology, having as a theme the method of learning anatomy by using concept mapping in 1st year students of the University of Medicine and Pharmacy, Cluj-Napoca, Romania.

## 2. Theoretical Foundation

Initially, on a previous meta-analysis [1], was observed that the use of conceptual maps has an effect on students’ memory in most medical studies but varies according to the subject studied. At the same time, the use of the conceptual maps over a longer period of time has led from insignificant to important results in the students’ short-term memory. Within this meta-analysis, however, only two studies on anatomy and none on the implication of conceptual maps for long-term memory were highlighted.

The current study starts from the established idea of continuous mental change of information through a constant process of elaborating this information [2,3,4,5]. In this way, the learning power could be modified by the quality of example development [6] or by the quality of personal explanations [7] arising from basic principles and less from superficial, short-term ones. In the following, we will mention some of the best-established theories regarding these learning and memorizing processes and the changes undergone to adapt to new situations.

The theory of cognitive overload, developed by Sweller, is based on the existence of 2 components in the cognitive elaboration process [8,9], one of permanent storage in long-term memory (LTM) and another of opposing their storage through working memory (WM). Thus, by reducing the impact of WM through the use of various tools (in this case conceptual maps), LTM would be improved.

Regarding WM, some authors consider it as actually activating LTM [10] or attached to the information processing system [11], while others with similar approaches to WM oppose LTM [12]. This LTM opposition comes as a confirmation of the magic number 7 [13] through which our own minds can simultaneously evaluate approximately 7 new units or chunks within the memory span.

In the mental lexicon (keyword theory) have been attempts to trace nets, in a similar way to conceptual maps, starting from a word placed at the level of a node, with similar words, thus creating a complex word-net [14,15].

These have led to classification theories into semantic memory [16] but also at the linguistic level [17], with the development of the node structure theory [18].

Concept mapping can thus be used, as a streamline tool. In order to genuinely generate learning, any piece of information must be passed through the filter of one’s own thinking, in order to verify its authenticity in a logical manner. There are teachers who only value the information as such [19], which is an important process but not sufficient in the long term for the information to be stored or used [19,20], which also implies its understanding [21,22]. This type of learning is more difficult to achieve and is based on integrating new concepts into existing ones [23,24,25,26,27], which is why a prior evaluation of both the students and their metacognitive abilities is necessary.

By introducing certain tools, the student can be determined to structure their ideas, by graphic or abstract representation [28,29], thus generating a learning process based on understanding the notions [30].

Such a tool is concept mapping, which arranges and connects the new concepts to the previous ones, for easier storage. They are also known as a “Novakian map” [31], having as a drawback, however, the non-polarization of the hierarchy described. That is why concept mapping must be carried out according to a qualitative model with the involvement of 3 important patterns—1. the words that describe the concept (with simple associations), 2. chaining (performing a correct hierarchy) and 3. Structuring the network (with interconnection, reflecting the hierarchy and complex interactions), which also denotes the understanding of the information [32,33].

Concept maps were introduced by Novak and Gowin in 1984 [34]; later, in 1993, Horton described them [35] as an “instructional tool” and in 2008, Gonzales as a pedagogical tool [36] in a study on physiology among medical students. In 2013, Bergman, de Bruin, et al. then studied students’ perceptions of anatomy [37] by a method very similar to the conceptual maps, the one achieved through problem-based learning, obtaining promising results in this case as well. In 2006, Canas et al. carried out an impressive meta-analysis [38] regarding the use of conceptual maps, reviewing the areas where the conceptual maps had or did not have efficiency; in 2016, in a review of the specialized literature summing up 132 studies, Balaid suggested future research directions [39] as well. Through all these studies and meta-analyses, it has been observed that in order to be effective, this method must be rigorously and correctly applied. Careful planning and implementation result in increasing the power of understanding the concepts as well as the relationships between concepts, thus promoting ideation and brainstorming and the fields of application of the method being drawn up, too. One of the most used fields remains the educational one but there are disciplines to which applicability remains limited.

Through this metacognitive tool associated with WM, represented by concept mapping, there is a recall of the cognitions included in the LTM in general but with an emphasis on hierarchies and relationships between concepts. Language can help in understanding but for a true knowledge of the complex relations between concepts, the student must be stimulated to “see” deeply, beyond words.

## 3. Background and Objectives

Given the existing theoretical basis, the working hypothesis was drawn:

H1-students in the experimental groups (learning from the concept mapping method) have different school performances than those in the control groups (who have learned by the traditional method).

As far as the objectives of this study are concerned, they are:

1. To observe whether there are differences regarding the memorization of the notions of anatomy, at the time of the examination, between the students who studied according to the method of concept mapping vs. the traditional method.
2. To analyze whether the language of learning (same or different from the first language) is involved in learning process through the concept mapping method. That is, in terms of language, which students would benefit more from this learning method.
3. What are the advantages of the method of study after a longer period of time, once the students have got used to the new method and continue to learn in the same way, by testing them at the time of the exam in the second semester.
4. To analyze whether there are differences between the 2 groups by testing long-term memory (every 6 months) after exam time, in both sections.
5. To study other factors that could moderate or mediate the results.

## 4. Methodology

The study involved 1st year students of the General Medicine Faculty “Iuliu-Hatieganu” University of Medicine and Pharmacy in Cluj-Napoca, Romania who were studying anatomy. The experimental group students were learning by using the concept mapping method, while the control group used the traditional method. They were subsequently examined through written exams.

### 4.1. The Participants, Research and Publication Ethics

The present study used as inclusion criteria first-year students in the anatomy laboratories. For this purpose, two series of students from the Romanian line of study and 3 series of students from the English line of study were selected to participate in the study, accounting for a total of 505 students from the 2015–2018 academic years.

The exclusion criteria were represented by the failure to sign the agreement to participate to the study, the integration of the students into a complementary year or the case of having more than 5 absences, considering that in these cases the students did not benefit from any of the learning methods. Students who had less than 5 absences in one semester, were included in the study for that semester.

The demographic dispersal of participants could be seen in Table 1.

All the students from the English line of study along with 16 others from the Romanian line had a different language of learning from their first language.

The participants filled out a form expressing their agreement to be part of the study and thus agreeing to the fact that some personal data could be used according to the law, respectively according to European Union (EU) Regulation, no. 679 of 27 April 2016 [40], on “the protection of natural persons with regard to the processing of personal data and on the free movement of such data” and the protocol was approved by the Ethics Committee of *“Iuliu Hatieganu”* University of Medicine (no. 9/15.01.2020). The students were randomly divided into 2 groups, an experimental and a control group for both the Romanian and the English line of study.

### 4.2. Data Collection

Several types of data collection were used to evaluate students’ knowledge, all of which were conducted only by written tests.

The first of these was represented by the grades obtained by the students when being tested in the written exam for each semester; the test contained multiple choice questions and the grades for the practical examination were eliminated. The last testing was performed approximately 6 months later, without students’ prior knowledge of the testing. This was carried out during class hours through unannounced testing and for this reason, the number of students tested, was smaller.

The results from the practical examination were eliminated from the study, in order to remove the subjective factor existing in this type of examination.

### 4.3. Research Design

We undertook a quasi-experimental design between subjects. The 489 students admitted into the study came from 2 different lines of study, the English and the Romanian. A random selection of study groups was chosen to participate in the Experimental group, the rest of the groups composed the Control group. The present study involves convenience sampling, all the participants being students at the Faculty of General Medicine. In this way, four groups resulted, both experimental groups used the concept mapping method during learning, according to the procedure, while both control groups benefited only from the classical method of learning.

The dependent variables are represented by the grades or number points obtained at the time of the exam, respectively in the test given 6 months later.

In addition to this, other moderating or mediating variables that might influence the outcomes, such as those reported in some studies, that is, gender [41,42] or age but also others (as number of absences, etc.), will be discussed.

### 4.4. Learning Method

The difference between the experimental and the control groups is given by the learning method, irrespective of the language of teaching, so in the following we will refer to the aspects of its implementation in the 2 groups.

During the course, the number of hours allotted to teaching anatomy were identical for both types of groups, all students attending the same courses, the difference consisting only in the laboratory lessons. In the experimental groups, in addition to the traditional working method, concept mapping was also discussed; the students prepared the concept maps at home, through individual work or sometimes in groups.

Concept mapping was not introduced from the beginning but only 4 weeks after the beginning of the academic year, this period being necessary both for the evaluation of the students regarding specific notions of anatomy and for a monitoring of the metacognitive abilities of each of them, in order to find out whether or not the degree of difficulty of the concept mapping method was suitable for each student. 

Also, during this time, the students were explained how and what aspects are to be considered when drawing concept maps, taking into account the following principles:

- developing a question or target topic (generally provided by the teacher),
- developing 10–20 words (concepts) put into boxes, considering their hierarchy,
- drawing explanatory arrows between concepts, first hierarchically, then through other associations.

This concept mapping had to include both anatomical and general notions, in order to make comparisons or to explain relations between these different concepts and could be accompanied by a concomitant or subsequent presentation and a relevant anatomical drawing, sections or sketch. Concept maps were drawn either on sheets of paper from the students’ drawing book or on a computer, using Cacoo software. The task was performed at home, individually by the students or in a group with designated colleagues, the respective work representing one of the topics that were to be discussed during the next stage. Thus, within each of these stages, a number of 2–3 topics were discussed, after which the presentations were subjected to a discussion within the groups. In this way, each student had to prepare a presentation every 3 weeks, so that, in the other stages, they could participate together with their colleagues in the discussions on the concept maps presented.

In the case of the control group, the students had as assignments the theoretical study of the specific notions, as well as of the anatomical sections or boards, without having to design concept maps. During the laboratory hours, the time allotted to discussing the theoretical notions was used according to the traditional method, respectively by the teacher presenting and explaining the notions of anatomy corresponding to the respective stage of the course.

For both groups, developing and presenting practical notions of the traineeship were performed similarly, much like in the total number of hours allocated to the students for studying anatomy during the course and laboratories.

All students who had a maximum of 5 absences were admitted to the study, the number of these being noted next to the students’ grades.

### 4.5. Statistical Analysis

Qualitative data were described numerically, by number and percentage. Quantitative data were described by mean and standard deviation, respectively graphically by means graphs. To test for differences between two independent groups of quantitative data, the Student test (t) was used for independent samples with equal or unequal variances. Equality of variances was assessed using the Levene test. To evaluate whether there are differences between several independent groups of quantitative data, the one-way ANOVA test was used. To evaluate the influence of moderation variables, besides variables of interest in evaluating differences between independent groups, the two-way ANOVA test was used, including for the interaction between the two variables. For all tests, the value of 0.05 was used as the significance threshold and the bilateral p value was taken into account in the tests that offered it. For statistical processing, the IBM SPSS software for computing and graphics version 25.0. was used.

For the Student-*t* test GPower 3.1.9.4. was used to calculate the difference between the mean values of the two independent groups. The input parameters were p-value (alpha) = 0.05, power = 0.95, effect size (d) = 0.5, allocation ratio N2(control group)/N1(experimental group) = 2.5, respectively Tail(s) - one. It turned out that the sample size should be at least 210 for both groups. Then, 2-way ANOVA test, for both sections and groups, with the same input parameters was made. It turned out that the sample size should be at least 400.

## 5. Results

A total number of 489 students were taken into consideration, that is, 185 at the Romanian line of study and 304 at the English line of study. Of them, 12 students were excluded from the study either because of their unwillingness to take part in it or due to school dropout; thus we ended up with 183 and 292 students, respectively.

A first verification was performed to test the internal validity of the study. We found a Cronbach alpha for each of the lines of study of 0.803 and 0.819, as well as in total 0.801, for all items in the questionnaire.

### 5.1. Results for Each Line of Study

We compared the marks taken by students in each semester (first and second) and the 6 months evaluation, between groups (English line experimental and control, Romanian line experimental and control) and then we compared the experimental with controls inside the English line and in the Romanian line (see Table 2). The means obtained in the experimental groups were superior to those of the control groups in both lines and for all examinations. All the differences were statistically significant, except for the Romanian line in the first semester. In the same table can be seen that the null hypothesis is rejected for almost all cases.

In addition, it was found that, in both lines, there were more absences among students in the experimental groups than in the control groups. So, for the Romanian line of study the mean number of absences was 2.07 for the experimental vs. 0.94 for the control group in the 1st Semester, respectively 3.07 vs 1.70 between the same groups in the 2nd Semester. For the English line of study, the same relation was 1.86 vs 1.41 in the 1st Semester, respectively 1.75 vs. 1.53 in the 2nd Semester.

### 5.2. Results according to the Language of Study

To see extent to which the language of study (same or different from the first language) could have an influence on the cognitive processes of the students, the whole group was divided into 4 groups, one experimental and one control group for each line of study. Subsequently, the students’ performance was tested by the 2-way ANOVA testing.

There were 169 students, whose language of study was the same as their native language, tested in the 1st semester, 167 in the 2nd semester and 109 were tested 6 months later. In the case when the language of study was different from the native language, 311 students were tested in the 1st semester, 317 in the 2nd semester and 147 students were tested 6 months later.

When testing the effects between subjects (Table 3), a significant relation was found (*p* < 0.05) given by both variables, group or language and students’ grades, without a significant interaction between these 2 variables (*p* > 0.05).

The data (Table 4) show the existence of a difference in favor of the experimental group if the language of study was the same as the first language and a greater difference if the language of study was different.

**Figure 1:**
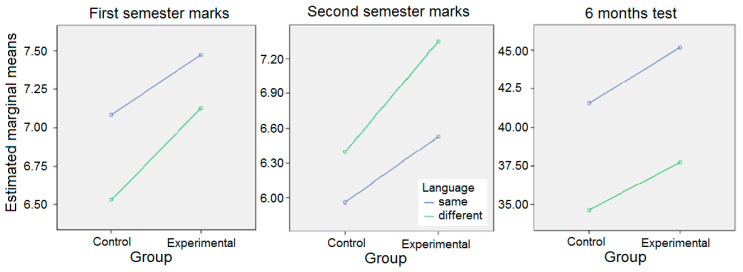
Comparing grades by language/group.

These differences can be seen in Figure 1 for each semester and the testing 6 months later.

### 5.3. Results Recorded according to the Participants’ Gender

Results recorded in the 1st Semester, 2nd Semester and at 6 months at both line of study, are as follows (Table 5):

In all these cases, as it can be seen in Table 5, there is an insignificant relation given by gender variable (*p* > 0.05). Testing with both variables, group and gender, also reveals an insignificant interaction (*p* > 0.05).

Table 6 shows the presence of a difference in favor of the experimental groups in all of the cases, greater for males than for females, even though this difference is not significant.

## 6. Discussions

The present study has as its main objective to determine whether and to what extent anatomy learning by using concept maps has an influence on the students in the 1st year of General Medicine, compared to the classical method, in the case of both those who studied in their first language and those who had a different language of study than their native language.

### 6.1. Discussions on Working Hypotheses

The mode of testing was the same for all the groups and it was performed by using the ANOVA method, which offered significant values, below the threshold value of 0.05, for all cases. With regard to the tests for the Romanian and English lines of study, in both cases, the t-Student test was performed on independent samples. Apart from the results for the first semester and at the borderline for the second semester, both in the case of the Romanian line of study, in all other cases, the null hypothesis was rejected.

The problem that arises at this time, is to see why in the case of the 1st semester grades, the null hypothesis could not be rejected. A first explanation is given by the fact that, as we have shown, in the first semester there is a stage of evaluating the students’ metacognitive capacities but also of learning how to create concept maps, which takes about 4–5 weeks, that is, about 1/3 of the entire semester; during this period the learning method was the same in both experimental and control groups. A second explanation is given by the language of study, in the sense that it generally enables a more efficient memorization of cognitions if it is the same as the first language. However, within the Romanian line of study, 91.5% of the students had the same language of study as their first language, which causes it to have an influence on short-term memory both in the control group and in the experimental group, diminishing the differences in cognitive results given by the method of studying. In the second semester, the method of learning with the use of concept maps is employed for the entire duration of the semester, thus the only difference is given by the language of study, just as in the case of testing 6 months later, where the null hypothesis for both sections is rejected.

These data show the way in which students respond to the new method of study, by the existence of a learning curve. Besides, Aliyari’s study on learning with concept maps vs. lecture in cardiac arrest [43] does not achieve significant results and is in contradiction with other studies by Dong et al., regarding the application of concept maps on the promotion of learning and interpretation of electrocardiogram [44] or Cutrer et al., regarding the patients’ diagnostic rates of pulmonary problems [45]. The reason for obtaining different results in these studies seems to be given (as Alyari found out) by the time allocated to study according to the method of concept maps—4 hours (in the case of the students who did not know anything about this method) in Aliyari’s study, respectively 10 h in the other studies. Indeed, Aliyari mentions that—“It seems that the use of concept mapping method requires more time to change the knowledge score.” (page 43). All these data are in concordance with the results of the meta-analysis by Nicoara et al. [1], where it is described that in order to obtain significant results with this method, at least 3.5 h are to be allotted to the new working method. This is where the less significant results are included, that is, the results obtained by the students who used concept mapping in the first semester as compared to the second semester.

### 6.2. Discussions on the Results by Lines of Study

At the level of the Romanian line of study, an increase in the differences between the experimental group and the control group can be noticed, starting with the results of the first semester until the 6-month testing. For the English line of study, where all students had a different first language, statistically significant differences were recorded in all cases. Most studies based on concept maps having explored only their impact or role on students and too little taking into account the learning curve of the method or testing its efficiency on long-term memory. Roessgera et al., in a qualitative study [46] on concept mapping, suggest that this method “is a learned skill that improves with repeated opportunities for practice and feedback” (page 20) but it would also improve the relation between concepts. For medical students, Torre et al. [47] associate this learning method with the improvement of critical thinking, while Chand et al. [48] appreciate the method as being useful both for teaching and learning, as we found by our research.

Absences were analyzed by their mean number. Both during the first and the second semesters, there was a greater number of absences in the experimental groups than in the control groups for both lines of study and yet their results were superior. Consequently, only the method of study is involved in the results obtained, the method of teaching is not. The explanations for this phenomenon may therefore lie either at the level of adaptability of this method to the students’ learning and thinking style or may be extracurricular, relating to motivation or reducing the anxiogenic phenomenon through this learning method.

### 6.3. Concept Mapping—Antibiotic or Vaccine?

Due to the results obtained 6 months after the implementation of the method, whether the study language was the same or different, we could conclude that this method of study is especially effective with long-term memory. It would probably also be of interest to study a possible change in the students’ behavior in relation to the learning process when this method is no longer effectively used, to see whether there has been a change in their process of analyzing the information, even in an unconscious manner. That is, when giving up the actual work with these maps, will students continue to make multiple correlations or not, so is there a change in their style of learning or thinking? In other words, it is worth researching whether these students will continue to work mentally, involuntarily, through the method of concept mapping, even if the actual descriptive work has ceased.

Hence, the following question arises—is concept mapping an antibiotic or a vaccine? That is, does this method take effect when effectively implemented (as an antibiotic) or has late effects, when it is no longer used but has produced changes in the mind (just like a vaccine does in the body).

### 6.4. Discussions on Mediating Factors

As in the present study there could not be found relevant data according to the age of the subjects, there being a difference of 4 years between the vast majority of the students and only a few cases being located on a level that exceeds 10 years, the research could not be continued in this regard.

Regarding language, it was found that there was a constant difference in favor of the experimental groups, whether the language of learning was the same or different from the first language. Another interesting aspect is that both experimental groups have managed to achieve favorable results, with a higher percentage of students with high grades and a smaller percentage of students with low grades but in different ways. Thus, in the experimental group that studied in a language different from the first language, there was an initial sharp increase in the number of students with high grades (in the first semester) and a further sharp improvement of those with low grades (in the second semester) and vice-versa for the groups that studied in the same language as the first language. These results should be compared with those in the literature but only few data were found regarding learning through concept maps in the same vs. different language from the native one. Marriott & Torres [49] show that students experienced limitations in expressing their ideas when they had to do it in a foreign language. These limitations were reported as being felt in oral communication with other colleagues as well. However, they considered this experience as an opportunity to put into practice their own ideas and prove their speaking skills. It is also shown that this way, students are encouraged to focus more on the connections between concepts (the meaning) than on the final product.

In the 2-way ANOVA testing, having as independent variables the group (experimental or control) and the language of study (different or the same as the first language), a statistically significant relation is noticed (*p* < 0.05) given by each variable and students’ grades in every semester, as well as in the 6-month testing. If the presence of interference between the variables, even if statistically insignificant, is detected in the semester tests, these interferences disappear completely at 6-months testing.

The same 2-way ANOVA testing but having as independent variables the gender and the group, yielded favorable results in experimental groups regardless of the line of study or gender, without a statistical influence of the gender on the grades. Even so, these differences between working groups are greater in both males and females in the semester testing in both lines of study but are reduced in the 6-month testing. These data suggest that the concept mapping method of learning has an effect on both sexes but men are more adaptable to it. The results from the literature are contradictory in this regard, some studies, such as that of Otor [41], find that females would benefit more from this method of learning, while Cheema & Mirza [50] show that the method would be more advantageous for the males.

## 7. Conclusions

To conclude, learning with the concept mapping method proves to be efficient especially in the case of the long-term memory regarding the study of anatomy in first-year students.

Also, in the short term, the results are statistically significant for subjects who have a different language of study than their first language, while for those with the same language of study as their first language, the results grow from statistically insignificant in the first semester to statistically significant at the 6-month testing.

At the same time, it can be concluded that language is involved not only in obtaining the aforementioned results but also in the way the method of learning with concept maps acts in time on students with different levels of preparation. So, this method of learning is efficient from the beginning in the case of students who are not so well-prepared, if their language of study is the same as their first language and respectively in that of well-prepared students, if their language of study is different from their first language.

As a conclusion, most of the students are influenced by this method of study in a positive way.

Considering the number of absences, the results have shown that, in spite of their better grades, the students in the experimental groups had more absences than those from the control groups. This leads to the conclusion that the method of learning influences the achievements and not the teaching method, as proven by the results of the present study.

Another conclusion is that the language of study (different or the same as the first language) makes its influence felt on both short-term and long-term memory. Thus, at least in the geographical area where the study was conducted, if the language of study is the same as the first language, the learning and memorization process will be easier for both the experimental and the control group. This explains the superior results obtained by these groups in the 6-month testing, compared to the groups that had a different language of study than the first language.

One of the limitations of the present study is, however, the impossibility of finding relevant data related to the age of the sample, since a very small percentage is actually in a different age range. Another limitation is the fact that the number of students could not be the same for the three examinations, either due to the number of absences made during the semester or to the absence on the actual examination. Moreover, as the study was conducted on a specific sample of students, the results cannot be generalized to the whole population and may differ from the results of similar studies but carried out on different samples.

The current study paves the way for other studies of learning through the method of conceptual maps in the medical field, being interesting and explaining the manner in which the concept mapping learning method achieves its efficiency. Thus, future studies, such as the implication of motivational, learning and thinking styles or changes in critical thinking when learning with the concept mapping method, would probably explain their mode of action.

On the other hand, it would be interesting to see what might happen in terms of student behavior in the learning process when removing this “trigger”.

What is noticeable from a practical point of view, however, is that by encouraging students, at least general practitioners, to use these tools or learning techniques, they will be able to improve their memory of anatomy. Thus, the student can find his associations alone, either between certain keywords or between concepts.

## Figures and Tables

**Table 1:** The demographic dispersal of participants.

Group		Control	Experimental	*p*-Value
Total				
Age (years), mean (SD)		20.28 (1.04)	20.25 (0.95)	0.789
Gender, n (%)	M	153 (45.9)	73 (46.2)	0.985
	F	180 (54.1)	85 (53.8)	
Examination, n	1’st Sem	332	148	
	2’nd Sem	333	151	
	6 Month	174	82	
English line of study				
Age (years), mean (SD)		20.39 (1.24)	20.32 (1.05)	0.695
Gender, n (%)	M	100 (52.4)	50 (44.25)	0.146
	F	91 (47.6)	63 (55.75)	
Examination, n	1’st Sem	190	105	
	2’nd Sem	191	110	
	6 Month	94	44	
Romanian line of study				
Age (years), mean (SD)		20.13 (0.65)	20.07 (0.59)	0.718
Gender, n (%)	M	53 (37.4)	23 (51.1)	0.105
	F	89 (62.6)	22 (48.9)	
Examination, n	1’st Sem	142	43	
	2’nd Sem	142	41	
	6 Month	80	38	

**Table 2:** Comparative results between groups per lines of study.

Grades/Test	Line of Study	Group (n)	Mean (std. dev.)	Low. Grades % (4–6)	High Grades % (8–10)	*p* Value *	*p* Value 4 Groups
**Sem. 1**	**Rom.**	Control (142)	6.99 (1.485)	33.8	37.3	0.055	0.004
		Experim. (43)	7.47 (1.077)	18.6	55.81		
	**Engl.**	Control (190)	6.57 (1.855)	33.16	18.42	0.020	
		Experim. (105)	7.11 (2.035)	28.57	36.19		
**Sem.2**	**Rom.**	Control (142)	5.88 (1.631)	57.04	16.9	0.050	<0.001
		Experim. (41)	6.44 (1.517)	48.78	31.71		
	**Engl.**	Control (191)	6.48 (1.986)	35.6	16.75	<0.001	
		Experim. (110)	7.42 (1.848)	17.27	33.63		
**After 6**	**Rom.**	Control (80)	41.45 (5.105)	37.5	23.75	0.001	<0.001
**Month**		Experim. (38)	44.71 (4.484)	10.53	38		
	**Engl.**	Control (94)	34.45 (4.550)	48.94	25.53	0.002	
		Experim. (44)	37.30 (5.733)	27.27	52.27		

Note: the results are colored in green (if are higher in the Experimental than Control group) or in red (if these are lesser); Sem. = Semester; Rom. = Romanian line; Engl. = English line; n = number; std. dev. = standard deviation; Std. Err = Standard error; Low. = Lower; Experim. = Experimental; *p* value * = comparisons between the experimental and control group, for each line of study and examination.

**Table 3:** Two-way ANOVA tests for effects of group, language and interaction between them.

Source	Sem I *p*-Value	Sem II *p*-Value	6 Months *p*-Value
Group	0.010	<0.001	<0.001
Language	0.019	0.002	<0.001
Language + Group	0.589	0.343	0.699

**Table 4:** Two-way ANOVA results by language/group.

Language	Group	1st Semester			2nd Semester			6-Month Testing		
		Median Mark	95% C. I.		Median Mark	95% C. I.		Median Mark	95% C. I.	
			Lower	Upper		Lower	Upper		Lower	Upper
Same	Control	7.084	6.786	7.382	5.962	5.647	6.277	41.566	40.442	42.690
	Experim.	7.474	6.921	8.027	6.528	5.927	7.128	45.182	43.476	46.887
Different	Control	6.532	6.292	6.773	6.396	6.143	6.650	34.643	33.653	35.633
	Experim.	7.127	6.802	7.452	7.348	7.012	7.684	37.735	36.335	39.134

Results are in blue if the language of study is the same with first language and in green if it is different (as in Figure 1).

**Table 5:** Two-way ANOVA test for effects of group, gender and interaction between them.

	Sem I *p*-Value		Sem II *p*-Value		6 Months *p*-Value	
	English	Romanian	English	Romanian	English	Romanian
Group	0.012	0.053	<0.001	0.051	0.002	<0.001
Gender	0.241	0.386	0.470	0.431	0.406	0.516
Gender + Group	0.181	0.191	0.293	0.242	0.699	0.098

**Table 6:** Two-way ANOVA results by gender/group.

Gender	Group	1st Semester		2nd Semester		6-Month Testing	
		English	Romanian	English	Romanian	English	Romanian
Women	Control	6.589	7.034	6.700	5.921	34.239	42.164
	Experimental	6.871	7.190	7.383	6.150	36.714	44.211
Men	Control	6.550	6.925	6.287	5.811	34.646	39.880
	Experimental	7.465	7.727	7.460	6.714	37.826	45.211
